# Implantable Electrical Stimulation at Dorsal Root Ganglions Accelerates Osteoporotic Fracture Healing via Calcitonin Gene‐Related Peptide

**DOI:** 10.1002/advs.202103005

**Published:** 2021-10-28

**Authors:** Jie Mi, Jian‐Kun Xu, Zhi Yao, Hao Yao, Ye Li, Xuan He, Bing‐Yang Dai, Li Zou, Wen‐Xue Tong, Xiao‐Tian Zhang, Pei‐Jie Hu, Ye Chun Ruan, Ning Tang, Xia Guo, Jie Zhao, Ju‐Fang He, Ling Qin

**Affiliations:** ^1^ Musculoskeletal Research Laboratory Department of Orthopedics & Traumatology Innovative Orthopaedic Biomaterial and Drug Translational Research Laboratory Li Ka Shing Institute of Health Sciences The Chinese University of Hong Kong Hong Kong Hong Kong 999077 China; ^2^ Shanghai Key Laboratory of Orthopaedic Implants Department of Orthopaedics Shanghai Ninth People's Hospital Shanghai Jiao Tong University School of Medicine 639 Zhizaoju Road Shanghai 200011 People's Republic of China; ^3^ Department of Biomedical Engineering The Hong Kong Polytechnic University Hung Hom 999077 Hong Kong; ^4^ Departments of Neuroscience and Biomedical Sciences City University of Hong Kong Kowloon Tong 999077 Hong Kong

**Keywords:** bone regeneration, CGRP, dorsal root ganglions, electrical stimulation

## Abstract

The neuronal engagement of the peripheral nerve system plays a crucial role in regulating fracture healing, but how to modulate the neuronal activity to enhance fracture healing remains unexploited. Here it is shown that electrical stimulation (ES) directly promotes the biosynthesis and release of calcitonin gene‐related peptide (CGRP) by activating Ca^2+^/CaMKII/CREB signaling pathway and action potential, respectively. To accelerate rat femoral osteoporotic fracture healing which presents with decline of CGRP, soft electrodes are engineered and they are implanted at L3 and L4 dorsal root ganglions (DRGs). ES delivered at DRGs for the first two weeks after fracture increases CGRP expression in both DRGs and fracture callus. It is also identified that CGRP is indispensable for type‐H vessel formation, a biological event coupling angiogenesis and osteogenesis, contributing to ES‐enhanced osteoporotic fracture healing. This proof‐of‐concept study shows for the first time that ES at lumbar DRGs can effectively promote femoral fracture healing, offering an innovative strategy using bioelectronic device to enhance bone regeneration.

## Graphical abstract

Electrical stimulation (ES) at dorsal root ganglions (DRGs) triggers calcium influx and activates the Ca^2+^/CaMKII/CREB signaling cascade, resulting in the transcription of calcitonin gene‐related peptide (CGRP). After synthesized in DRGs, CGRP is transported to peripheral nerve terminals and released following depolarization by ES. The released CGRP and subsequently increased type‐H vessel formation promote osteoporotic fracture healing.

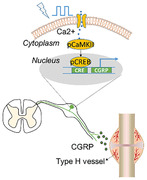

## 1 Introduction

Osteoporotic fracture is a common disease in clinical practice,^[^ ^1^ ^]^ while its treatment is still a challenge due to the compromised bone regeneration and vessel formation.^[^ ^2^ ^]^ The prolonged healing period results in higher morbidity, disability, and even mortality, especially in elder patients.^[^ ^3^ ^]^ Various strategies aiming at enhancing either osteogenesis or angiogenesis have been developed to rescue the impaired osteoporotic fracture healing,^[^ ^4^, ^5^, ^6^ ^]^ while the outcome remains unsatisfactory.^[^ ^3^, ^7^, ^8^ ^]^

Bone tissues are innervated by a dense network of sensory nerves, and the most generally distributed sensory nerves are calcitonin gene‐related peptide (CGRP) positive nerves.^[^ ^9^, ^10^, ^11^ ^]^ CGRP positive nerves regenerate themselves during fracture healing.^[^ ^12^, ^13^ ^]^ At the terminals of these nerves, CGRP is released following neuronal depolarization and then exerts its biological functions including angiogenesis and osteogenesis in bone regeneration.^[^ ^14^, ^15^, ^16^ ^]^ Impaired release of CGRP at fracture sites retards fracture healing, leading to delayed fracture union and even nonunion,^[^ ^17^ ^]^ while local supplementation of CGRP enhances the bone regeneration in bone defect.^[^ ^14^, ^15^, ^18^, ^19^ ^]^ Thus, CGRP is essential for fracture healing and may be a target for fracture healing enhancement.

To constantly improve CGRP at fracture site, the most common way is to develop a drug delivery system carrying CGRP protein. Recently, gelatin microspheres and strontium–calcium phosphate cement have been tested as carriers for CGRP.^[^ ^20^, ^21^ ^]^ However, due to the technical restriction, the degradation of recent materials is difficult to match the bone ingrowth during healing.^[^ ^3^ ^]^ In addition, as a peptide without quaternary structure of protein, CGRP degrades rapidly in plasma, which limits the application of exogenous CGRP in bone healing.^[^ ^14^, ^22^ ^]^ The endogenous CGRP is synthesized in dorsal root ganglion (DRG) and its transcription is highly regulated by depolarization.^[^ ^23^ ^]^ We previously found that voltage‐controlled square unipolar pulse could increase the expression of CGRP in DRGs,^[^ ^24^ ^]^ suggesting electrical stimulation (ES) at lumbar DRGs may increase endogenous CGRP for fracture healing.

In the present study, we selected specific ES parameters for promoting the biosynthesis and release of CGRP and investigated the underlying mechanisms in in vitro studies and then verified the selected parameters in osteoporotic rats. We next engineered a bioelectronic system with special soft electrodes and evaluated its therapeutic effect in a rat femoral osteoporotic fracture model. Meanwhile, the mechanisms on how ES at DRGs accelerates fracture healing were investigated as well.

## 2 Results

### 2.1 Decreased CGRP Expression Was Associated with Impaired Fracture Repair in Osteoporosis

**Figure 1:**
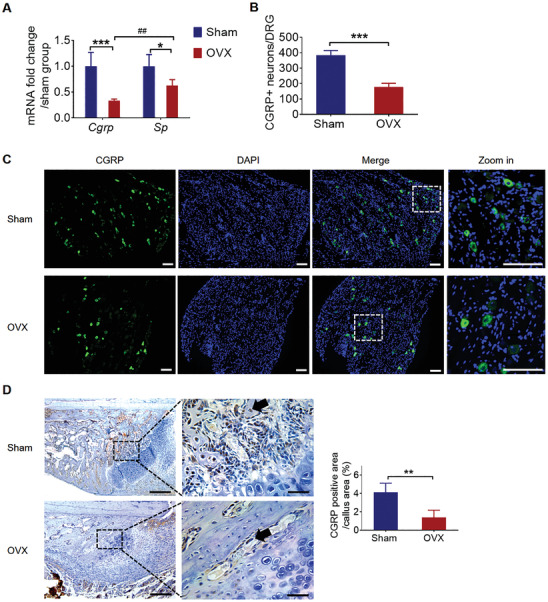
Decreased CGRP expression in DRGs and callus during osteoporotic fracture healing. A) The mRNA level of *Cgrp* and *Sp* in DRGs at week 2 in sham and OVX group (mean ± SD, two‐way ANOVA with Bonferroni tests, *P < 0.05 and ***P < 0.001 comparison between sham and OVX group, unpaired Student's *t*‐test, ^##^ *P* < 0.01 comparison between CGRP and SP in OVX group. *n* = 4 per group per time point). B) Quantification and C) representative images of CGRP positive neurons in DRGs at week 2 in sham and OVX group (mean ± SD, unpaired Student's *t*‐test, ***P < 0.001, *n* = 5 per group). Scale bar: 100 µm. D) Representative images and E) quantification of CGRP positive area (black arrows) in fracture callus at week 2 in sham and OVX group (mean ± SD, unpaired Student's *t*‐test, **P < 0.01, *n* = 5 per group). Scale bar: 50 µm (left row) and 200 µm (right row).

To assess the sensory neuronal activity during osteoporotic fracture healing, we first induced osteoporosis in 6‐month‐old female rats by ovariectomy (OVX) and established closed femoral fracture according to our previous protocol.^[^ ^25^ ^]^ As shown by radiographs, the fracture callus was smaller in OVX group as compared to that of the sham group (Figure S1a, Supporting Information). Micro‐computed tomography (micro‐CT) also showed the bone volume/tissue volume (BV/TV) and bone mineral density (BMD) of TV in OVX group was significantly lower than that of the sham group (Figure S1b, Supporting Information). Moreover, the OVX group exhibited lower maximum load at week 4 as compared to the sham group (Figure S1c, Supporting Information). We then isolated DRGs to evaluate and compare the sensory neuronal activity at week 2 between sham group and OVX group. Quantitative polymerase chain reaction (qPCR) results showed that both CGRP and substance P (SP) decreased in OVX group as compared to sham group, but the decline of CGRP was more obvious than SP (**Figure** **1**a). Histological analysis further confirmed the low expression of CGRP in DRGs (Figure 1b,c) and fracture callus (Figure 1d,e) in OVX group. To further investigate whether CGRP expression decline was associated with the impaired fracture healing, we directly injected 100 × 10^−9^ m CGRP at the fracture sites for the first 2 weeks after fracture. The micro‐CT showed that CGRP supplementation significantly promoted callus formation (Figure S2a,b, Supporting Information) with increased BV and BV/TV at week 4, although no significant difference was found in TV between OVX + NC (negative control: the solvent without CGRP) group and OVX + 100 × 10^−9^ m CGRP group (Figure S2c–e, Supporting Information). These results indicate that CGRP may be the potential target for enhancing osteoporotic fracture healing.

### 2.2 ES Upregulated CGRP Synthesis and Triggered Its Release In Vitro

**Figure 2:**
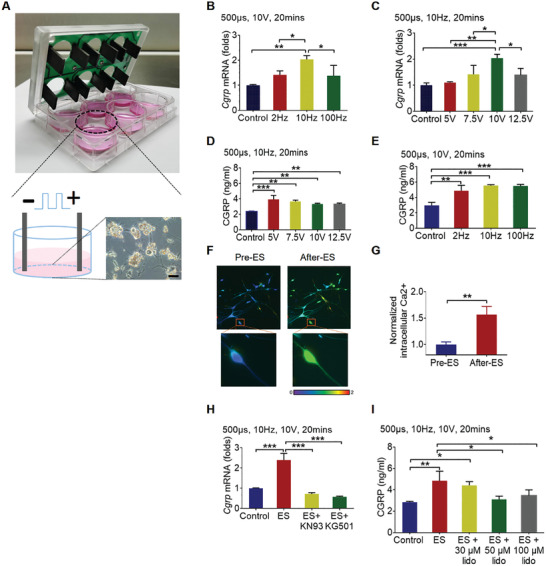
ES upregulated CGRP biosynthesis and triggered its release in vitro. A) The ES setup for DRG neurons. DRG neurons were seeded on laminin and poly‐d‐lysine hydrobromidecoated 6‐well plate, and electrical pulse was generated by C‐Pace stimulator. *Cgrp* mRNA in neurons and CGRP protein in culture medium were quantified immediately after stimulation. B–E) *Cgrp* mRNA and CGRP protein level after ES with different frequencies and voltages (mean ± SD, one‐way ANOVA with Tukey's tests, *P < 0.05, **P < 0.01, and ***P < 0.001, *n* = 3 per group). F) Intracellular Ca^2+^ was monitored by laser confocal microscopy with the fluorescent Ca^2+^ indicator Fluo‐4 AM. G) The intracellular Ca^2+^ after stimulation, normalized to that before stimulation (mean ± SD, unpaired Student's *t‐*test, **P < 0.01, *n* = 3 per group). H) *Cgrp* mRNA and I) CGRP protein level after ES with indicated drugs treatment (mean ± SD, one‐way ANOVA with Tukey's tests, *P < 0.05, **P < 0.01, and ***P < 0.001, *n* = 3 per group).

To sort out the optimal ES parameters for upregulating CGRP expression, we used voltage‐controlled square unipolar pulses (pulse width: 500 µs) with different frequencies and voltages to stimulate the DRG neurons isolated from 4‐week‐old female rats (**Figure** **2**a). The ES‐induced *Cgrp* mRNA level could be adjusted by altering the frequency (Figure 2b) and voltage (Figure 2c). The released CGRP after ES did not depend on the frequency within the range of 2–100 Hz (Figure 2d) or the voltage between 5 and 12.5 V (Figure 2e). To provide a sustainable CGRP source for fracture healing enhancement at early stage, we chose the parameters (10 Hz, 10 V, 500 µs) that could stimulate both biosynthesis and release of CGRP. Calcium imaging showed that the selected parameters induced a notable increase of intracellular calcium (Ca^2+^) in the neurons (Figure 2f,g). qPCR results showed that both KN91 (Ca^2+^/CaMKII inhibitor) and KG501 (CREB inhibitor) significantly inhibited ES‐triggered *Cgrp* production (Figure 2h). Meanwhile, immunofluorescent staining showed the percentages of pCaMKII and pCREB positive cells among CGRP positive cells were significantly higher in the ES group as compared to the control group (Figure S3a,b, Supporting Information). These results suggested that ES upregulated CGRP expression by activating Ca^2+^/CaMKII/CREB signaling pathway. We next added lidocaine into culture medium to block action potential and found that the released CGRP after ES was dependent on the fired action potential (Figure 2i). In addition, the release of CGRP was independent on the intracellular Ca^2+^ as BAPTA (intracellular Ca^2+^ chelator) failed to abrogate ES‐induced CGRP release (Figure S3c, Supporting Information).

### 2.3 Design and Verification on the Effectiveness of Bioelectronic System

**Figure 3:**
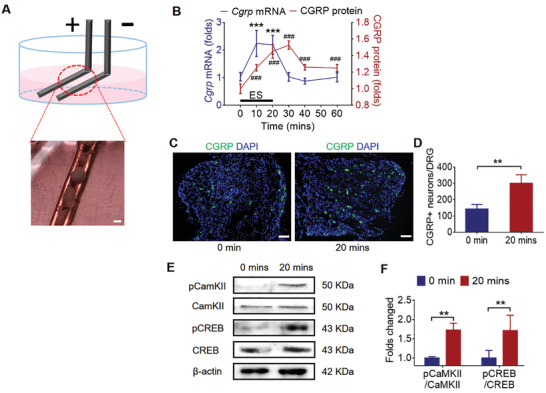
Design and verification of the implantable electrodes in vitro. A) Schematic of the stimulation setup for DRG tissue. The DRGs isolated from osteoporotic rats were placed between the two paralleled platinum electrodes, and electrical pulse stimulation was applied through the electrodes. Scale bar: 2 mm. B) The dynamic CGRP expression at mRNA and protein level was quantified after ES (normalized to 0 min, mean ± SD, one‐way ANOVA with Tukey's tests, ***P < 0.001 as compared to the mRNA level before ES, ^###^ *P < *0.001 as compared to the protein level before ES, *n* = 4 per group). C,D) Representative images and quantification of CGRP positive neurons in DRGs at 0 and 20 min after ES (mean ± SD, unpaired Student's *t*‐test, **P < 0.01, *n* = 4 per group). Scale bar: 50 µm. E,F) Western blot analysis of pCaMKII, CaMKII, pCREB, and CREB expression in DRGs after ES (normalized to 0 min, mean ± SD, two‐way ANOVA with Bonferroni tests, **P < 0.01, *n* = 4 per group).

To utilize the ES‐induced CGRP biosynthesis and release for fracture healing enhancement, implantable and direct‐contact electrodes are designed and fabricated (see the Experimental Section). First, we designed two custom‐engineered electrodes and directly electrostimulated the DRGs isolated from osteoporotic rats through these electrodes (**Figure** **3**a). qPCR results showed that 10 min ES significantly upregulated the CGRP transcriptional level by twofold, and this effect persisted during the 20 min period and then vanished (Figure 3b). ELISA analysis showed that CGRP concentration in DRGs increased immediately after ES and peaked at 30 min after stimulation (1.5‐fold higher than the control), and declined after 60 min stimulation where the value was still significantly higher relative to the control (Figure 3b). The increased CGRP expression in DRGs was further confirmed by immunofluorescent staining (Figure 3c,d). Meanwhile, the activation of Ca^2+^/CaMKII/CREB signaling pathway after ES was verified by western blot analysis (Figure 3e,f).

**Figure 4:**
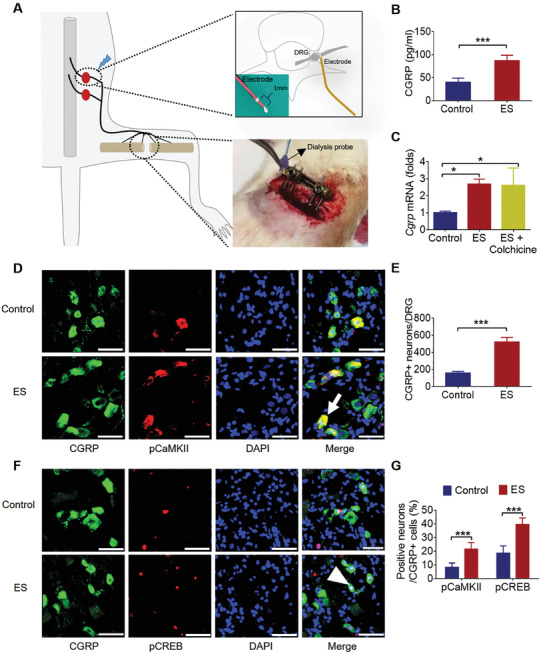
ES at DRGs promoted the synthesis and release of CGRP in vivo. A) Schematic of the stimulation setup in vivo and the dialysis system for collecting protein from fracture site. Two soft electrodes were implanted at L3 and L4 DRGs and a bone defect was created at femur for inserting a dialysis probe. B) CGRP concentration in the bone defect regions of control and ES group (mean ± SD, unpaired Student's *t*‐test, ***P < 0.001, *n* = 4 per group). C) *Cgrp* mRNA levels in the DRGs of the control, ES, ES + colchicine group (mean ± SD, one‐way ANOVA with Tukey's tests, *P < 0.05, *n* = 4 per group). D,F) Representative immunofluorescent staining showing the colocalization of CGRP positive neurons with pCaMKII (white arrow) and pCREB (white arrowhead) in the DRGs with or without ES treatment. Scale bar: 50 µm. E,G) Quantification of CGRP positive neurons and the percentages of pCaMKII positive and pCREB positive in CGRP positive neurons of ES group as compared to that of the control group (mean ± SD, unpaired Student's *t*‐test for (E), two‐way ANOVA with Bonferroni tests for (G), ***P < 0.001, *n* = 5 per group).

We next built up an implantable bioelectronics system with the soft electrodes and tested its in vivo effectiveness in osteoporotic rats (**Figure** **4**a). After determined the exact DRGs that innervate femur by retrograde tracer (Figure S4a,b, Supporting Information), we implanted soft electrodes at L3 and L4 DRGs in rats and delivered ES for 20 min. After ES, DRGs were isolated to evaluate the CGRP transcription level and the dialysate at femoral defect region was collected for determining the CGRP concentration (Figure 4a). ELISA analysis showed that ES significantly increased the CGRP concentration at femoral midshaft by twofold (Figure 4b). To investigate the source of released CGRP, ES was first applied at the spinal nerve that was dissected next to DRGs and then at the DRGs with lidocaine infiltration (Figure S5a,b, Supporting Information). ES at the dissected spinal nerve induced a similar CGRP release to ES at DRGs, while lidocaine infiltration significantly blocked the ES triggered CGRP improvement (Figure S5c, Supporting Information). In addition, ES did not affect the axonal transportation of CGRP (Figure S6, Supporting Information), ruling out the possibility that the released CGRP were from the store in spinal nerves. These results indicated that the released CGRP induced by ES was a rapid secretion from the vesicular stored in nerve terminals. Apart from the released CGRP, ES upregulated the *Cgrp* mRNA level in DRGs by twofold and this positive effect persisted even when the axonal transportation was blocked by colchicine (Figure 4c). This result suggested that the increased *Cgrp* expression was directly stimulated by ES, rather than the feedback post CGRP release. Consistent with our in vitro results, immunofluorescent analysis showed that ES significantly increased CGRP positive neurons and increased the pCaMKII and pCREB activity among CGRP positive DRG neurons (Figure 4d–g).

### 2.4 ES at DRGs Enhanced Osteoporotic Fracture Healing

**Figure 5:**
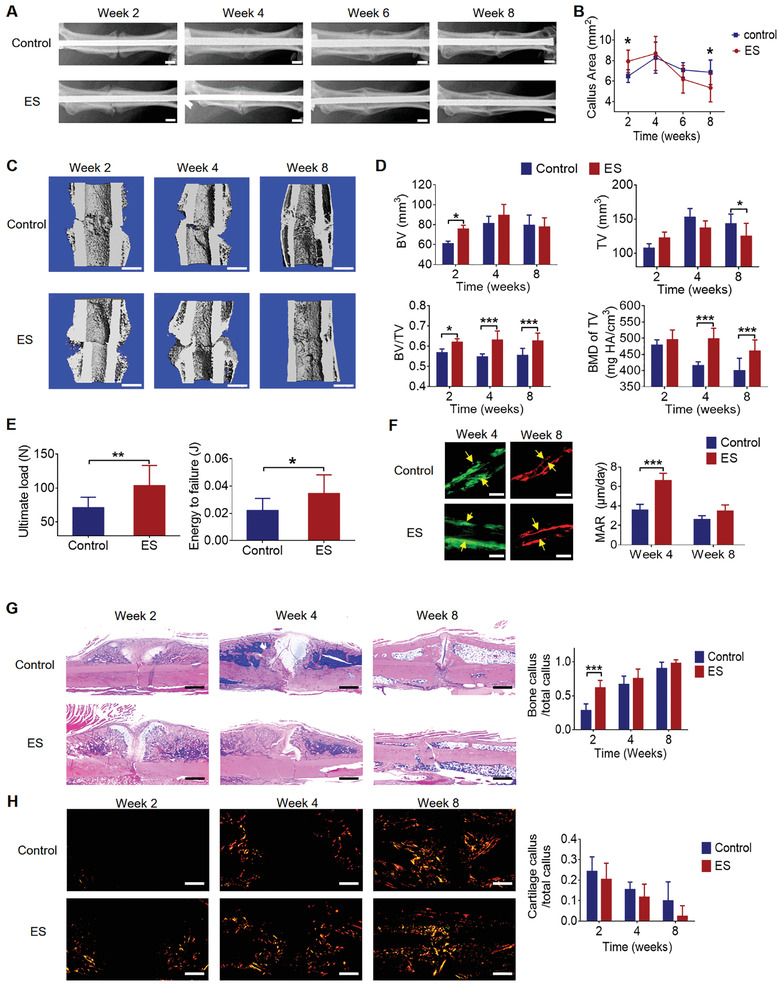
ES at DRGs accelerated osteoporotic fracture healing. A) Representative radiographs of fractured rat femora from ES group and the control group. Scale bar: 2 mm. B) Quantification of the callus area (mean ± SD, two‐way ANOVA with Bonferroni tests, *P < 0.05, *n* = 10 per group). C) Micro‐CT 3D reconstruction and D) quantitative measurements of TV, BV, BV/TV, and BMD of TV at week 2, 4, and 8 in control and ES group (mean ± SD, two‐way ANOVA with Bonferroni tests, *P < 0.05 and ***P < 0.001, *n* = 5 per group at week 2 and 4, *n* = 10 per group at week 8). Scale bar: 2 mm. E) Ultimate load and energy to failure of the fractured rat femora at week 8 in the control and ES group (mean ± SD, unpaired Student's *t*‐test, *P < 0.05 and **P < 0.01, *n* = 10 per group). F) Calcein green/xylenol orange labeling and comparison of MAR between ES and the control group at week 4 and 8 (mean ± SD, two‐way ANOVA with Bonferroni tests, ***P < 0.001, *n* = 5 per group). Scale bar: 5 µm. G) H&E staining and quantification of bone and cartilage fraction in callus at week 2, 4, and 8 in ES and the control group (mean ± SD, two‐way ANOVA with Bonferroni tests, ***P < 0.001, *n* = 5 per group). Scale bar: 1 mm. H) Representative images of polarized light at week 2, 4, and 8 in ES and the control group. Scale bar: 1 mm.

We next investigated the therapeutic effect of ES at DRGs on osteoporotic fracture healing. Soft electrodes were implanted at L3 and L4 DRGs and daily ES (20 min per day) was given for the first 2 weeks after fracture. Radiographs showed that ES group had significantly larger callus at week 2 which became smaller at week 8 as compared to the control group, while there was no significant difference between the two groups at week 4 and 6 (**Figure** **5**a,b). Micro‐CT results showed that the BV and BV/TV in the ES group were significantly higher than the control group, while no significant difference was found in TV between the two groups at week 2 (Figure 5c,d). The BV/TV of the ES group remained higher than that of the control group at week 4 and 8 (Figure 5d). The TV of the ES group was no different at week 4, but lower at week 8, than that of the control group (Figure 5d). The BMD of TV of fractured rat femora at week 4 and 8 in ES group was significantly higher than that of the control group (Figure 5d). Consistently, the expression of osteocalcin (OCN) and Sp7 transcription factor (SP7) in facture callus was higher in the ES group than in the control group at week 2 and 4, with no difference between the two groups at week 8 (Figure S7, Supporting Information). These results suggested that ES group had larger and denser callus at the early stage of fracture haling, and might have a faster remodeling process at the later stage, as compared to the control group. In addition, biomechanical test at week 8 demonstrated that ES group had significantly higher ultimate load and energy to failure as compared to the control group (Figure 5e).

Histologically, the mineral apposition rate (MAR) of the ES group was significantly higher than that of the control group at week 4, while there was no difference between the two groups at week 8 (Figure 5f). Hematoxylin and eosin (H&E) staining showed that ES group had more newly formed bone at week 2, and better morphology with smaller callus at week 8 as compared to the control group (Figure 5g). Polarized light images revealed that ES group had more and brighter fibers in the fracture callus at week 2 and 4, and had a better alignment of the collagen fibers in the newly formed bone at week 8, as compared to the control group (Figure 5h). These results indicated that the bone formation was enhanced post ES treatment and the remodeling was accelerated at later stage of fracture healing, which was in line with our callus measurement and micro‐CT results.

Histological analysis at week 2 after fracture showed that the CGRP expression in DRGs and fracture callus was significantly higher in ES group as compared to the control group (Figure S8, Supporting Information). The increased CGRP concentration in DRGs and callus after ES was also observed in non‐osteoporotic fracture (Figure S9, Supporting Information). As CGRP commonly participated in nociceptive pathways in the peripheral nervous system, pain‐related behaviors were monitored and pain levels were measured by electronic von Frey in both ES and control groups from day 3 to day 14 after fracture (Figure S10a,b, Supporting Information).^[^ ^26^, ^27^ ^]^ There was no significant difference in pain‐related behaviors including spontaneous guarding and flinching (Figure S10c, Supporting Information), and paw withdrawal threshold (Figure S10d, Supporting Information). Because spinal microglial activation is involved in pain hypersensitivity,^[^ ^28^ ^]^ we also stained a microglia‐specific marker, ionized calcium‐binding adapter molecule‐1 (IBA‐1), in the spinal dorsal horn (L4) at week 2. Again, the immunofluorescent staining showed that no significant difference in IBA‐1 intensity between ES and the control group (Figure S11, Supporting Information). Therefore, the enhanced CGRP expression by daily ES for 2 weeks induces neither pain behavior nor pain hypersensitivity. In addition, this bioelectronics implant has good biocompatibility without systematic or local immune reaction (Figure S12, Supporting Information).

### 2.5 Indispensable Role of CGRP in ES Enhanced Fracture Healing

**Figure 6:**
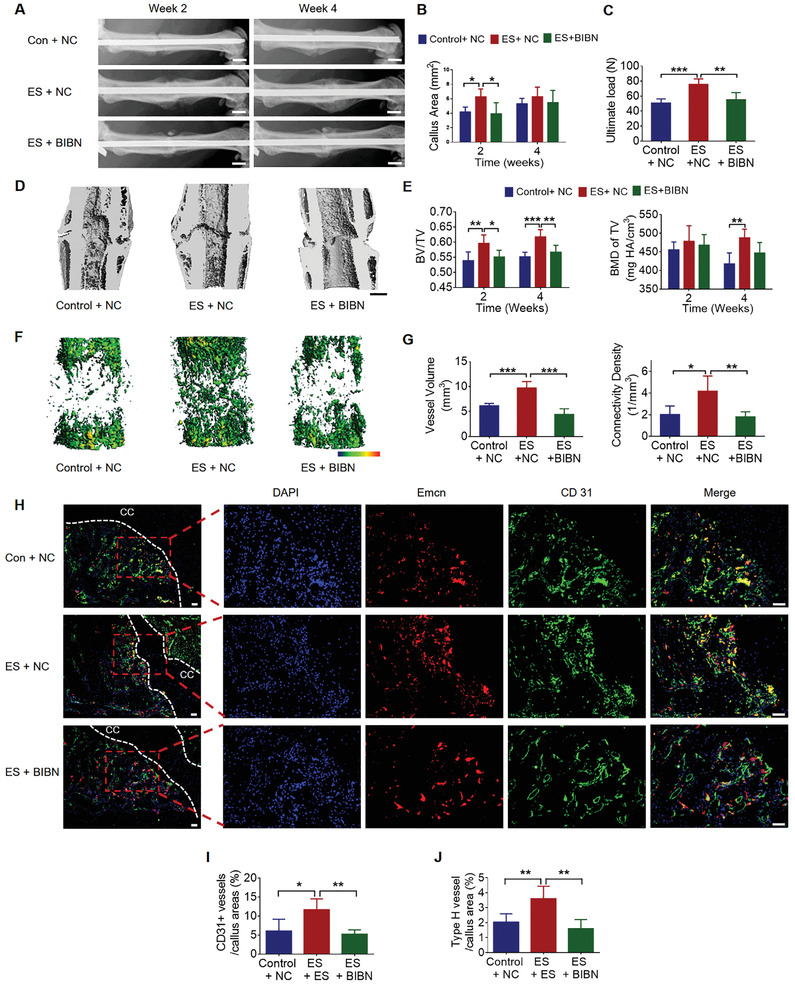
CGRP mediated type‐H vessel formation in ES enhanced fracture healing. A) Representative radiographs of the individual groups at week 2 and week 4. Scale bar: 2 mm. B) Quantification of the callus size (mean ± SD, two‐way ANOVA with Bonferroni tests, *P < 0.05, *n* = 5 per group). C) Ultimate load of the fractured rat femora at week 4 (mean ± SD, one‐way ANOVA with Tukey's test, **P < 0.01 and ***P < 0.001, *n* = 5 per group). D) 3D reconstruction and E) micro‐CT measurements of BV/TV and BMD of TV of the fractured rat femora at week 2 and 4 of individual groups (mean ± SD, two‐way ANOVA with Bonferroni tests, *P < 0.05, **P < 0.01, and ***P < 0.001, *n* = 5 per group). Scale bar: 2 mm. F) Representative 3D reconstructed images of the newly formed vessel around fracture line at week 2. G) Vessel volume and connectivity density were quantified by micro‐CT (mean ± SD, one‐way ANOVA with Tukey's test, *P < 0.05, **P < 0.01, and ***P < 0.001, *n* = 5 per group). H) Representative immunofluorescent double staining of CD31 and endomucin at week 2 after fracture. CC: cartilage callus. Scale bar: 100 µm. I) Quantification of CD31 positive vessel and J) type‐H vessel among fracture callus area (mean ± SD, one‐way ANOVA with Tukey's test, *P < 0.05 and **P < 0.01, *n* = 5 per group).

To investigate the role of CGRP in ES at DRGs for enhancing osteoporotic fracture healing, CGRP receptor antagonist (BIBN 4096BS, BIBN) was delivered to fracture site. Radiographic images showed significantly smaller callus in ES + BIBN group as compared to ES + NC (negative control: the solvent without BIBN) group (**Figure** **6**a,b). Biomechanical tests demonstrated that the ultimate load at week 4 in ES + NC group was significantly higher than that of the control + NC group and ES + BIBN group, respectively (Figure 6c). Micro‐CT results showed that the BV/TV was significantly higher in ES + NC group at week 2 and week 4 as compared to control + NC group (Figure 6d,e). ES + BIBN group had significantly lower BV/TV at week 2 and 4 as compared to ES + NC group (Figure 6d,e). Meanwhile, the TV density of fractured rat femora at week 4 in ES + NC group was significantly higher than that of the control + NC group (Figure 6d,e).

As shown in 3D reconstructed images, ES + NC group had larger vessel volume around fracture site than control + NC and ES + BIBN group at week 2 (Figure 6f). Quantitative analysis showed that the vessel volume in ES + NC group was significantly larger than that of the control + NC group and ES + BIBN group, respectively (Figure 6g). The connectivity density in ES + NC group was significantly higher than that of the control + NC group and ES + BIBN group (Figure 6g). We further performed double immunofluorescent staining for CD31 and endomucin (Figure 6h), recently identified as type‐H vessel.^[^ ^29^ ^]^ Similar to angiography, CD31 positive vessel area was significantly increased by ES and further blocked by BIBN (Figure 6i). ES significantly increased type‐H vessel formation in fracture callus and BIBN significantly blocked ES‐enhanced type‐H vessel formation (Figure 6j). These results indicated that the release of CGRP induced by ES at DRGs promoted fracture healing via type‐H vessel formation.

## 3 Discussion

In this study, we found that ES directly upregulated the synthesis of CGRP in DRGs via activating Ca^2+^/CaMKII/CREB signaling pathway and triggered a rapid CGRP release from the vesicular stores at nerve terminals via action potential. We next engineered implantable soft electrodes and implanted them at L3 and L4 DRGs for daily ES. ES at DRGs for 2 weeks upregulated CGRP biosynthesis and triggered its release at femoral region, and the released CGRP promoted type‐H vessel formation for osteoporotic fracture healing enhancement.

We previously used ES to modulate the biosynthesis of CGRP and demonstrated that ES at lumbar DRGs could prevented unloading induced bone loss.^[^ ^24^ ^]^ In this article, we further optimized the ES parameters for CGRP biosynthesis and release, and enlarged the application of ES at lumbar DRGs into osteoporotic femoral fracture healing enhancement, a more challenge condition than preventing unloading induced bone loss. As a commonly used physical stimulation, ES has been previously investigated for long bone fracture healing.^[^ ^30^ ^]^ However, our simulation method is different from previous documented ES methods for bone healing in terms of electrodes implantation and potential mechanisms. The electrical or electromagnetic field generated by previous ES methods is directly applied at the fracture site, modulating local biological functions for fracture healing. However, in our study, electrodes were implanted at lumbar DRGs which innervating the region of bone repair and the electrical impulse was directly delivered to the DRGs for CGRP synthesis and release. The released CGRP, acting as a mediator of ES, was expected to accelerate bone healing. Our results showed that ES at DRGs efficiently promoted fracture repair with advanced bone formation and biomechanical property, and without affecting pain level. In short, our study is a first proof‐of‐concept study showing that repeatable and controlled stimulation to DRGs by direct‐contract electrodes could be used for femoral fracture healing enhancement. Although direct‐contract electrodes are invasive, the successful verification of our innovative concept indicates that noninvasive neuromodulator including skin contact electrodes, electromagnetic filed and focused ultrasound shall be developed to boost CGRP‐mediated bone regeneration by reprogramming the cellular activity of DRGs.^[^ ^31^, ^32^ ^]^ Actually, as the gene transcription in DRGs can be reprogramed by different ES parameters^[^ ^33^ ^]^ and the synthesized neuropeptides have a variety of applications,^[^ ^15^, ^34^, ^35^ ^]^ our concept tested here is not limited to single‐gene expression instead has broader applications.^[^ ^36^ ^]^ For example, an electrical signal also controls brain‐derived neurotrophic factor synthesis in DRGs and its release benefits peripheral nerve regeneration.^[^ ^37^ ^]^

Another highlight of this study is that we have revealed the mechanisms underlying ES induced CGRP biosynthesis and release. We demonstrated that ES promoted CGRP expression in DRGs by activating Ca^2+^/CaMKII/CREB signaling pathway. Similarly, Yan et al. detected the activation of this signaling pathway after ES in cultured DRG neurons.^[^ ^38^ ^]^ Our present result is also consistent with a previous study reporting that the phosphorylation of CREB at Ser‐133 increases in proportion to electrical stimulus frequency between frequencies of 1 and 10 Hz.^[^ ^39^ ^]^ After synthesized in DRGs, CGRP is transported along sciatic nerve to nerve terminals and stored in the large dense vehicle, and then released via synaptobrevin‐I mediated exocytosis following neuronal depolarization.^[^ ^16^ ^]^ A previous study has demonstrated that ES at sciatic nerve increases the CGRP concentration in skin using a dialysis probe.^[^ ^40^ ^]^ Using a similar dialysis system,^[^ ^41^ ^]^ we directly measured the CGRP concentration at bone tissue and found that ES at DRGs increased CGRP concentration in femoral midshaft by twofold. To the best of our knowledge, we are the first group to identify the CGRP concentration change at bone tissue after ES. Of note, due to inherent technical problem, this finding may be confounded as the influence of the released CGRP from muscle^[^ ^42^ ^]^ and skin.^[^ ^40^ ^]^ In addition, we found that the released CGRP after ES was dependent on the fired action potential but independent of intracellular calcium. Action potential dependent secretion of CGRP has been well established in previous in vitro study,^[^ ^43^ ^]^ and the fired antidromic compound action potentials along peripheral nerve have been directly recorded after ES applied at spinal cord^[^ ^44^, ^45^ ^]^ and phrenic nerve.^[^ ^46^ ^]^ The existence of intracellular calcium independent vesicular secretion has also been observed in mammalian sensory neurons.^[^ ^47^ ^]^

After released at fracture site, CGRP was demonstrated to promote the formation of type‐H vessel which couples angiogenesis and osteogenesis. Increased type‐H vessel abundance is reported to significantly accelerate bone fracture healing and spinal fusion in previous studies.^[^ ^48^, ^49^ ^]^ The close location relationship between type‐H vessel and CGRP positive nerves has been identified during the ossification of endplates in painful disc model.^[^ ^11^ ^]^ This is also consistent with previous reports that CGRP has dual biological functions in terms of angiogenesis^[^ ^18^ ^]^ and osteogenesis.^[^ ^14^, ^25^ ^]^ However, the underlying mechanisms how CGRP increases type‐H vessel formation remains unclear, which shall be investigated in future study. In addition, as CGRP can directly affect osteoclastogenesis and osteoclasts play a crucial role in callus remodeling, osteoclasts may also engage in ES‐enhanced fracture healing.^[^ ^14^, ^50^ ^]^

CGRP is described to distribute in nociceptive pathways in peripheral nervous system and its receptors are also expressed in pain pathways.^[^ ^51^ ^]^ However, consistently with our present study, previous studies showed that increased local CGRP concentration at fracture site neither elevated the pain level nor induced aberrant sprouting of nerve fibers which can also cause pain.^[^ ^25^ ^]^ Weidner et al. also found that local application of high concentration of CGRP (10^−5^ m) at skin did not induce any detectable pain behavior.^[^ ^52^ ^]^ This contradiction may be explained that the exact mechanisms of CGRP in nociceptive processing are not fully clarified^[^ ^53^ ^]^ and the fracture itself causes pain which may cover up the effect of increased CGRP.

Although most osteoporosis occurs in postmenopausal women, the prevalence of senile osteoporosis is increasing due to the aging of population.^[^ ^54^, ^55^ ^]^ Thus, it is necessary for us to investigate the therapeutic effects of ES at DRGs on fracture healing enhancement in aged male rats in future. In addition, some other nerves, such as SP or tropomyosin receptor kinase A positive nerves, may also respond to ES stimulation, which shall be investigated in future study.

## 4 Conclusion

In conclusion, the present study demonstrates that ES at lumbar DRGs can modulate the biosynthesis of CGRP in DRGs and its release to peripheral bone for osteoporotic fracture healing enhancement. The successful verification of our innovative concept inspires that non‐invasive neuromodulator shall be developed to reprogram the cellular activity of DRGs for peripheral bone regeneration.

## 5 Experimental Section

### Study Design

Parameters were first selected for upregulating the biosynthesis and release of CGRP and investigated the underlying mechanisms by in vitro and in vivo experiments. Then soft electrodes were engineered and were implanted at lumbar DRGs to investigate the therapeutic effect of ES at DRGs on osteoporotic fracture healing by micro‐CT, biomechanical test, angiography, and histological analysis, respectively. In fracture model, ES was daily delivered for the first two weeks and the amplitude was set at 80% of the motor threshold which was defined as the maximum voltage without triggering perceptible hind limb movement in awake rats as motor movement positively affects fracture healing.^[^ ^56^ ^]^ CGRP receptor antagonist (BIBN) was directly applied to fracture site to investigate the role of CGRP in ES at DRGs induced fracture healing enhancement. As BIBN was dissolved in saline with 2% HCL, the solvent without BIBN was also directly injected as negative control (NC) in Control + NC group and ES + NC group. All animal experimental protocols used in the present study were approved by the Animal Experimentation Ethics Committee of the Chinese University of Hong Kong (Ref No. 18‐254‐GRF).

### Electrical Stimulation of DRG Neurons and Tissues

Sensory neurons were isolated from the lumbar DRGs of 4‐week‐old female rats by sequential digestion using collagenase A (1 mg mL^−1^, Sigma‐Aldrich, St. Louis, MO, USA) according to reported protocols.^[^ ^25^ ^]^ The isolated cells were then resuspended with Neurobasal A medium containing 2% B‐27, 1% PSN, 1% glutamine, 20 ng mL^−1^ NGF, 10^−5^ m fluorodeoxyuridine and 10^−5^ m uridine and plated on coverslips (2.5 cm in diameter) coated laminin (20 ng mL^−1^) and poly‐d‐lysine hydrobromide (50 ng mL^−1^). The medium and supplements were all from Thermo Fisher Scientific Inc. After 1‐day growth, DRG neurons were electrically stimulated using the C‐Dish (Ionoptix, Dublin, Ireland) for 20 min with rectangular pulses. In treatment groups, KN94 (1 × 10^−6^ m, Shanghai Haoyuan Chemex press, Shanghai, China) and/or KG501 (10 × 10^−6^ m, Shanghai Haoyuan Chemex press, Shanghai, China) were added into culture medium and incubated for 30 min before ES.

The intact DRGs were isolated from osteoporotic rats and cultured with Neurobasal A medium. After 1‐day culture, DRGs were directly put between two custom electrodes and immersed with medium. The electrodes were connected to pulse generator and DRGs were stimulated with the same rectangular pulses (amplitude: 4 V cm^−1^, pulse width: 500 µs).

### Measurement of Intracellular Ca^2+^ in DRG Neurons

Intracellular calcium in DRG neurons was determined by fluo‐4 acetoxymethylester (Fluo‐4 AM) (Solarbio, Beijing, China) with a qualitative confocal laser microscope (Leica TCS‐SP5, DM6000‐CFS, Leica, Germany). In brief, neurons were incubated in Hanks’ balanced solution supplemented with 4 × 10^−6^ m Fluo‐4 AM and 1 × 10^−6^ m Pluronic F‐127 (Sigma‐Aldrich, St. Louis, MO, USA) for 30 min in the dark, and further incubated in Hanks’ balanced solution in absence of Fluo‐4 AM for 20 min at room temperature. The images of DRG neurons before and after ES were acquired using confocal laser microscope. Fluo‐4 AM was excited at 488 nm wavelength light. Fluorescence intensity was recorded through 525 nm bandpass filter and images were taken and stored before and after ES. The normalized intracellular Ca^2+^ was defined as the fold fluorescence over baseline after background subtraction.

### ELISA for CGRP Concentration

The proteins in DRG neurons were extracted with RIPA buffer containing protease inhibitors (Sango, Shanghai, China). The CGRP concentration in the dialysis sample and DRG neurons were measured with ELISA kit (BioVendor, Czech Republic) according to the established protocol.^[^ ^25^ ^]^

### Western Blot

The proteins of the intact DRG were isolated for western blot analysis using previous published protocol.^[^ ^57^ ^]^ The expressions of pCaMKII (1:100, 10011438, Thr286/Thr287, Cayman chemical, USA), CaMKII (1:1000, PA5‐39732, Thermofisher, USA), pCREB (1:1000, S133, ab32096, Cambridge, MA, USA), and CREB (1:1000, ab31387, Cambridge, MA, USA) in each sample were normalized to *β*‐actin using Image J software.

### Retrograde Tracing of DRGs Innervating Femur

Under general anesthesia (75 mg kg^−1^ Ketamine and 10 mg kg^−1^ Xylazine, intraperitoneal injection), a hole was created on the cortex of femur using 23‐gauge needle. Then FG (0.1 mg per rat, Polysciences Inc., Eppelheim, Germany) was injected through the hole. Three days later, bilateral L1–L6 DRGs were isolated and 10 µm thick cryosections were prepared to quantify the FG‐labeled neurons.

### Establishment of Closed Femoral Fracture in Sham and Osteoporotic Rats

To simulate postmenopausal osteoporosis which is the most common osteoporosis in clinical practice, 6‐month‐old female rats were subjected to OVX.^[^ ^25^, ^55^ ^]^ After 3 months, closed fracture was created on the right femur in sham and osteoporotic rats according to the well‐established protocol.^[^ ^25^ ^]^

### Radiographic Analysis of Fracture Callus

All rats at indicated time point were examined by digital X‐ray machine (MX‐20, Faxitron X‐Ray Corp., Wheeling, IL, USA) with voltage at 38 kV and current at 0.29 mA. Callus area was measured based on a lateral radiograph of each rat with Image J. The measurement for each callus was repeated three times, and the average was used for statistical analysis.

### Micro‐CT

Femora were subjected to *ex vivo* micro‐CT analysis (µCT40, Scanco Medical, Switzerland) with a voltage of 70 KeV and a current of 114 µA. After scanning, the images were reconstructed. Then BV (mm^3^), total TV (mm^3^), BV/TV fraction, and BMD were calculated for evaluating the quality of newly formed bone around fracture line using built‐in software.^[^ ^58^ ^]^ The region of interest was 7 mm around fracture line, and 150–1000 Hounsfield were defined as mineralized bone (Sigma = 1.2, Support = 2) following published protocols.^[^ ^25^, ^59^ ^]^

### Biomechanical Test

The biomechanical properties were evaluated by four‐point bending test using a mechanical testing machine (H25KS; Hounsfield Test Equipment Ltd., UK) with a 250 N load cell. The femora were loaded in anterior–posterior direction on lower supporting bars at 20 mm apart, and upper bars at 10 mm apart and the compression load was applied at a rate of 5 mm min^−1^ until failure. The ultimate load (N) and energy to failure (J) were obtained and analyzed.^[^ ^60^ ^]^

### qPCR

Total RNA was isolated from tissue or cells using TRIzol reagent (15596026; Invitrogen, Waltham, MA, USA). After determining concentration and purity by NanoDrop 2000 (Thermo Fisher Scientific, USA), RNA was reversely transcribed into cDNA using the First Strand cDNA kit (Takara, DaLian, China). Real‐time quantitative PCR reaction was performed with cDNA as template using TF pack power SYBR Green qPCR SuperMix‐ UDG.^[^ ^25^ ^]^ The 5′ and 3′ primers used were as follows: *Cgrp*, CTCAGCTCCAAGTCATCGCT and CTGCCATCTTCCTGGGTGATTT; *Sp*, ATGAAAATCCTCGTGGCGGT and ATCTGACCATGCCCAGCATC; *β‐actin*, GCAGGAGTACGATGAGTCCG and ACGCAGCTCAGTAACAGTCC. *β‐actin* was used as the internal control for normalization.

### Immunofluorescent Staining of DRGs and Spinal Cord

DRGs or spinal cord were isolated and fixed in buffered formalin solution overnight and then dehydrated in 30% sucrose for 24 h. Cryosections (10 µm thick) were prepared for following immunofluorescence staining according to previous protocol.^[^ ^18^ ^]^ The sections were incubated with primary antibodies overnight at 4 °C and then Alexa Fluor‐conjugated secondary antibodies were used to bind the indicated primary antibody and DAPI was used to stain the nuclei. The primary antibodies included CGRP (1:200; ab5694; Abcam, Cambridge, MA, USA), pCREB (1:200; S133; ab32096, abcam, Cambridge, MA, USA), pCaMKII (1:200; Thr286/Thr287; Cayman chemical, USA), and IBA‐1 (1:200, Wako, Japan). The stained sections were observed with a fluorescent microscope (Leica Q500MC, Leica, Germany). For CGRP positive neurons counting in DRG, five sections of each DRG were randomly selected and CGRP immunoreactive neurons with visible nucleus were counted and averaged by two independent investigators. For a single DRG, the CGRP expression was defined as the total number of CGRP stained neurons in five sections. For each sample, CGRP expression in DRG was defined as the average of CGRP expression in L3 and L4 DRGs. The fluorescent intensity of IBA‐1 staining was quantified by Image J software. Five sections of each spinal cord were randomly selected and dorsal horn was defined as the region of interest to derive a mean fluorescent intensity.

### Histological Analysis and Immunostaining of Fracture Callus

After micro‐CT analysis, the femur samples were decalcified with 12.5% ethylene diamine tetraacetic acid and then embedded in paraffin. Serial 5 µm sections of each sample were cut with a microtome (Leica RM2255, Leica, Germany). These decalcified sections were then stained with H&E for general histological evaluation and then quantified by Image J for comparing bone–tissue–area fraction. The distribution and arrangement of collagen in decalcified sections were evaluated by polarized light microscope (Leica Q500MC, Leica, Germany).

CGRP (1:200, ab5694, Abcam, Cambridge, MA, USA), SP7 (1:200; ab94744; Abcam,Cambridge, MA, USA), OCN (1:400; PA5‐78871; Thermo Fisher Scientific, Waltham, MA), and corresponding isotype controls (Figure S13, Supporting Information) were separately incubated in fracture callus. DAB substrate solution (ab64264; Abcam, Cambridge, MA, USA) was applied to reveal the color of antibody staining and hematoxylin was used to stain the nuclei. CD31 (10 *μ*g mL^−1^, AF3628, R&D system, USA) and Emcn (1:100, BS‐5884R, Bioss, China) was used to label type H vessel. Alexa Fluor‐conjugated secondary antibodies were then used to bind the indicated primary antibodies and DAPI was used to stain the nuclei. The positive area was counted at 4 standard selected high‐powered fields (HPF) (× 100) in each section. For each sample, 4 midsagittal tissue sections were evaluated. Positive area verse total callus was calculated.

### Local Application of CGRP or BIBN at Fracture Sites

Exogenous CGRP (100 × 10^−9^ m, ab47101, Abcam, Cambridge, MA, USA) dissolved in 100 µL protein stabilization (2% bovine serum albumin, 0.2% polyvinyl pyrrolidone, 0.2% NaCl, 20% sucrose, and 0.05% Sodium Azide, all from Sigma‐Aldrich, St. Louis, MO, USA) was directly injected at fracture site in OVX + 100 × 10^−9^ m CGRP group, and equal volume of protein stabilization was also injected in OVX + NC group. BIBN4096BS (BIBN, 300 µg kg^−1^ body weight, Shanghai Haoyuan Chemexpress, Shanghai, China) dissolved in saline with 2% HCL was separately injected to the fracture site in OVX + CGRP group, ES + BIBN group every day for the first 2 weeks after fracture.^[^ ^25^ ^]^ Saline with 2% HCL was also injected at fracture sites in ES + NC and the control + NC group. The BIBN, CGRP, or NC were daily injected via a 1 mL injector according to the previous protocol.^[^ ^25^ ^]^

### Bone Histomorphometric Analysis

Calcein green (10 mg kg^−1^, Sigma‐Aldrich, St. Louis, MO, USA) was injected intraperiotoneally at week 3 and week 4, and xylenol orange (30 mg kg^−1^, Sigma‐Aldrich, St. Louis, MO, USA) was injected intraperiotoneally at week 7 and week 8 (3 days before the sacrifice of animals). These samples were then embedded in methyl methacrylate and 10 µm sagittal sections were obtained according to the published protocol.^[^ ^25^ ^]^ Fracture callus was chosen as region of interest and the MAR was measured by OsteoMeasure system (OsteoMetrics Inc., Decatur, GA, USA).^[^ ^61^ ^]^

### ES System Implantation In Vivo

The electrode used in this study was fashioned from 2 silver plating wires (outer diameter: 0.3 mm, inner diameter: 0.18 mm; MFT‐S‐33, Hongan Company, China) with insulation removed at their terminals. The uninsulated terminal of one wire wrapped helically over the insulated portion of the other wire. The distance between the uninsulated terminals of two wires was 1 mm (Figure S14, Supporting Information). This approximately axially symmetric design provided bipolar contact around DRG independent of the rotational position. The electrodes were connected to stimulator (JDS‐2900 signal generator; JUNTEK; Zhengzhou Minghe Electronic Science and Technology Company, China) by coaxial cable (Figure S14, Supporting Information). Electrodes implantation was performed according to the previous published protocol.^[^ ^24^, ^62^ ^]^ In brief, after general anesthesia, right transverse processes of L3 and L4 were exposed through a dorsal incision, and 1 mm diameter hole was drilled at each transverse process. After inserted into the hole, the electrode was sutured to muscle to maintain its position (Figure S14, Supporting Information). Surgical incision was then sutured sequentially. Daily 20 min stimulation was delivered for the first two weeks after fracture.

### Bone Defect and Dialysis System

In brief, a 3 mm segmental bone defect was created on the midshaft of right rat femur and a monolateral external fixator was assembled to fix two segments with four stainless steel pins (Xinzhong Company, Tianjin, China) according to previous studies^[^ ^63^, ^64^ ^]^ for inserting a microdialysis probe (CMA Microdialysis AB, Solna, Sweden) with a cutoff threshold of 100 000 Da. The dialysis sample was collected on ice with perfusate (Ringer's solution with 1% bovine serum albumin) at a flow rate of 2 µL min^−1^. CGRP concentration of the dialysis sample was determined by ELISA kit.

A small incision was made at low back to expose L3 and L4 DRGs and then colchicine (25 × 10^−3^ m; HY‐16569; MCE, Monmouth Junction, USA) or lidocaine (2%; HY‐B0185; MCE, Monmouth Junction, USA) was directly applied around DRGs.^[^ ^65^, ^66^ ^]^

### Angiography

Under general anesthesia, the abdomen cavity of a rat was opened and contrast reagent (MICROFIL, MV‐117; Flowtech, Carver, MA, USA) was injected into abdominal aorta using the established protocol.^[^ ^67^, ^68^ ^]^ After vascular corrosion casts were obtained, the femora were dissected and decalcified with 9% formic acid. Then the decalcified femora were scanned by µCT (Scanco Medical, Switzerland) for visualizing vessels in three dimensions and analyzing vascular volume (mm^3^) and connectivity density (mm^−3^). The infused radio‐opaque substance was defined at a threshold of greater than 120 Hounsfield unit with a low‐pass Gaussian filter (Sigma = 1.2 and support = 2) following the published protocol.^[^ ^68^ ^]^

### Pain Assessments

Pain‐related behaviors including spontaneous guarding and flinching, and paw withdrawal threshold measured by electronic von Frey were evaluated at days 3, 7, and 14 after fracture. In brief, the rats were transported to experimental room and kept in separate cage with a perforated metal sheet for 1 h and this procedure was carried out after surgery for 3 days for habituation. At day 3, the rats were placed in cages for 30 min and then the time spent guarding or flinching over a 2 min observation period were recorded and averaged by two investigators who were blinded to the experimental condition of the animals.^[^ ^69^ ^]^ After that, paw withdrawal threshold was evaluated by electronic von Frey test (Electronic von Frey Anesthesiometer 2390, IITC, Inc., USA). For each rat, the tip of force transducer was vertically applied at footpad center through the mesh bottom and the operator gradually increased the pressure to induce a clear paw withdrawal response. The withdrawal threshold was recorded by the instrument.^[^ ^27^ ^]^

### Statistical Analysis

All data were expressed as the mean ± SD. After homogeneity test of variance, unpaired Student's *t* test (two‐tailed), or repeated ANOVA with Tukey's post hoc tests, or one‐way ANOVA with Tukey's post hoc tests, or two‐way ANOVA with Bonferroni post hoc tests were separately used in different experiments as indicated in the figure legend. Prism 6.01 (GraphPad Software Inc.) was used for statistical analysis and *P* < 0.05 was considered statistically significant.

## Conflict of Interest

The authors declare no conflict of interest.

## Supporting information

Supporting Information

## Data Availability

Research data are not shared.
