# Supplementary material for: Implantable Electrical Stimulation at Dorsal Root Ganglions Accelerates Osteoporotic Fracture Healing via Calcitonin Gene‐Related Peptide
Source: Adv Sci (Weinh). 2021 Oct 28;9(1):2103005. doi: 10.1002/advs.202103005 (PMC8728818; doi:10.1002/advs.202103005)
Supplement: Supplementary file 1 — Supporting Information [file ADVS-9-2103005-s001.pdf]

© 2021 Wiley-VCH GmbH

ADVANCED SCIENCE
Open Access

Supporting Information

for *Adv. Sci.*, DOI: 10.1002/advs.202103005

Implantable Electrical Stimulation at Dorsal Root Ganglions Accelerates Osteoporotic Fracture Healing via Calcitonin Gene-Related Peptide

*Jie Mi, Jian-Kun Xu, Zhi Yao, Hao Yao, Ye Li, Xuan He, Bing-Yang Dai, Li Zou, Wen-Xue Tong, Xiao-Tian Zhang, Pei-Jie Hu, Ye Chun Ruan, Ning Tang, Xia Guo, Jie Zhao**, Ju-Fang He*, and *Ling Qin**

Supporting Information

Implantable Electrical Stimulation at Dorsal Root Ganglions Accelerates Osteoporotic Fracture Healing via Calcitonin Gene-Related Peptide

Jie Mi[†], *Jiankun Xu*[†], *Zhi Yao*[†], *Hao Yao, Ye Li, Xuan He, Bingyang Dai, Li Zou, Wenxue Tong, Xiaotian Zhang, Peijie Hu, Ye Chun Ruan, Ning Tang, Xia Guo, Jie Zhao*[*], *Jufang He*[*], *Ling Qin*[*].

[†]These authors contributed equally to this work.

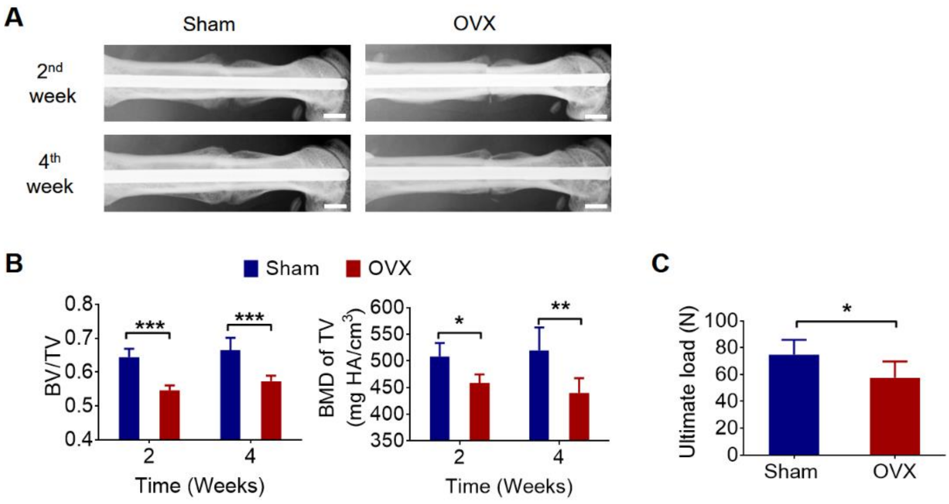

Figure S1. Fracture healing was impaired in osteoporosis

(**A**) Representative radiographs of rat fractured femora at week 2 and 4 in sham group and OVX group. Scale bar, 2 mm. (**B**) Micro-CT measurements of BV/TV and TV density of fractured femora at week 2, and 4 in sham and OVX group (mean ± SD, two-way ANOVA with *Bonferroni* tests, *$P < 0.05$, **$P < 0.01$, ***$P < 0.001$, $n = 5$ per group per time point). (**C**) Ultimate load of rat fractured femora at week 4 in sham and OVX group (mean ± SD, unpaired Student's t test, *$P < 0.05$, $n = 5$ per group).

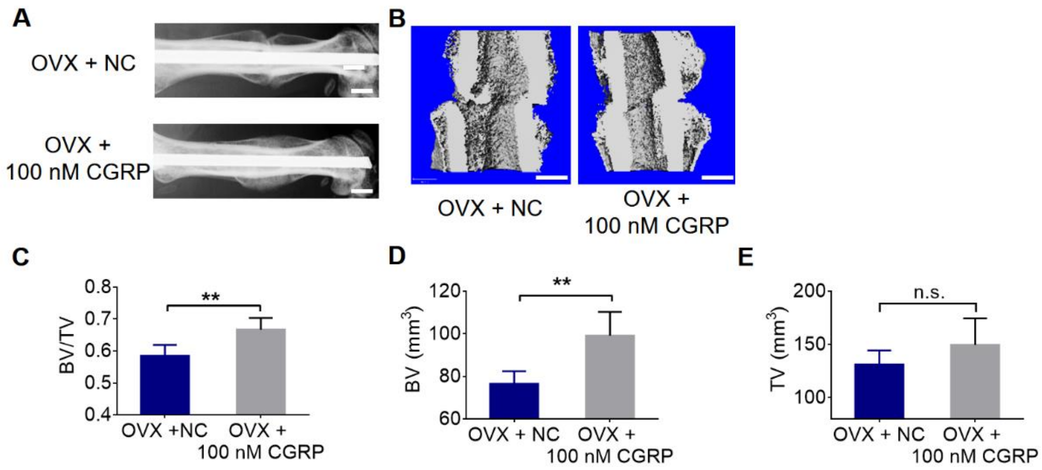

Figure S2. CGRP supplementation accelerated osteoporotic fracture healing.

(**A**) Representative radiographs and (**B**) 3D reconstruction of fractured femora at week 4 in OVX + NC group and OVX + 100 nM CGRP group. Scale bar, 2 mm. (**C** to **E**) Micro-CT measurements of BV, TV, and BV/TV of fractured femora at week 4 in OVX + NC as compared to OVX + 100 nM CGRP group (mean ± SD, unpaired Student's *t* test, $^{**}P < 0.01$, n.s. $P > 0.05$, $n = 5$ per group).

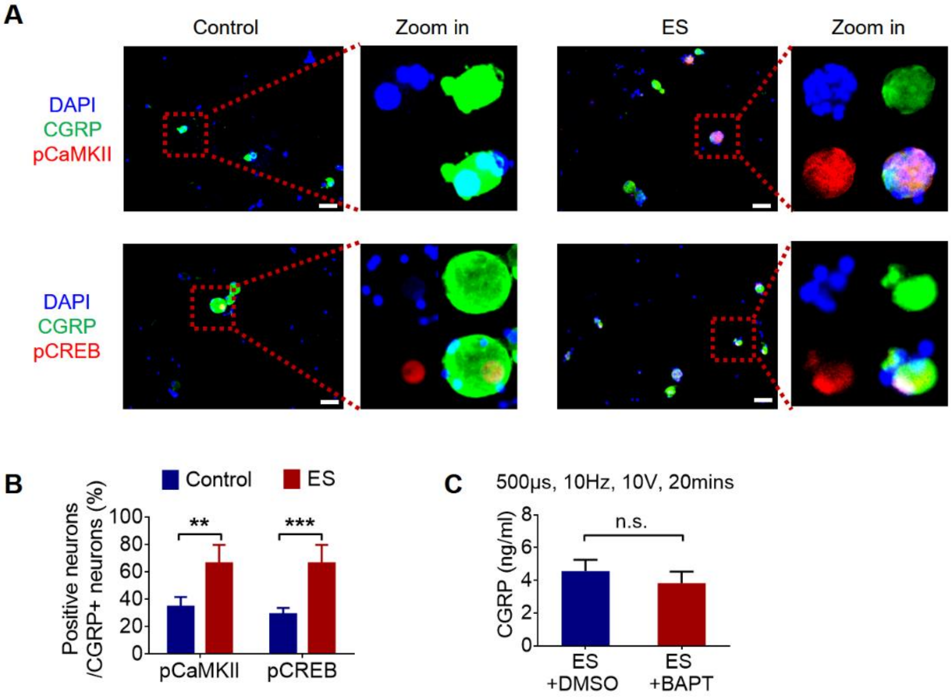

Figure S3. ES activated Ca^{2+}/CaMKII/CREB signaling pathway in DRG neurons and BAPT did not blocked ES induced CGRP release.

(**A**) Representative images of colocalization between CGRP, pCaMKII, pCREB in DRG neurons treated with or without ES. Scale bar, 50 μm. (**B**) Quantification of the percentage of pCaMKII positive and pCREB positive neurons among CGRP positive neurons in ES group as compared to control group (mean ± SD, two-way ANOVA with *Bonferroni* tests, $**P < 0.01$, $***P < 0.001$, $n = 4$ per group). (**C**) CGRP protein level after ES with indicated drugs treatment (mean ± SD, unpaired Student's *t* test, n.s. $P > 0.05$, $n = 3$ per group).

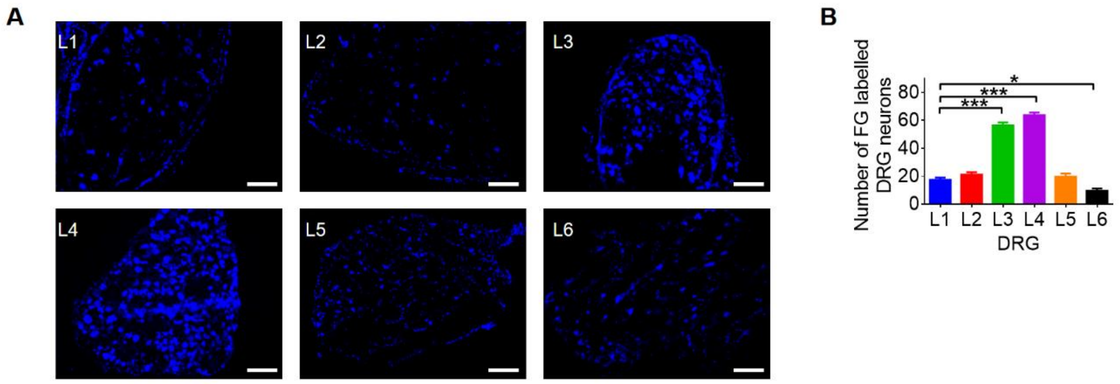

Figure S4. Femur was mainly innervated by L3 and L4 DRGs.

(**A**) Fluoro-gold (FG) labeled neurons in lumbar L1-L6 DRGs. Scale bar, 50 μm. (**B**) Quantification and comparison of FG labeled DRG neurons among lumbar L1-L6 DRGs (mean ± SD, one-way ANOVA with *Tukey's* tests, *$P < 0.05$, ***$P < 0.001$, $n = 5$).

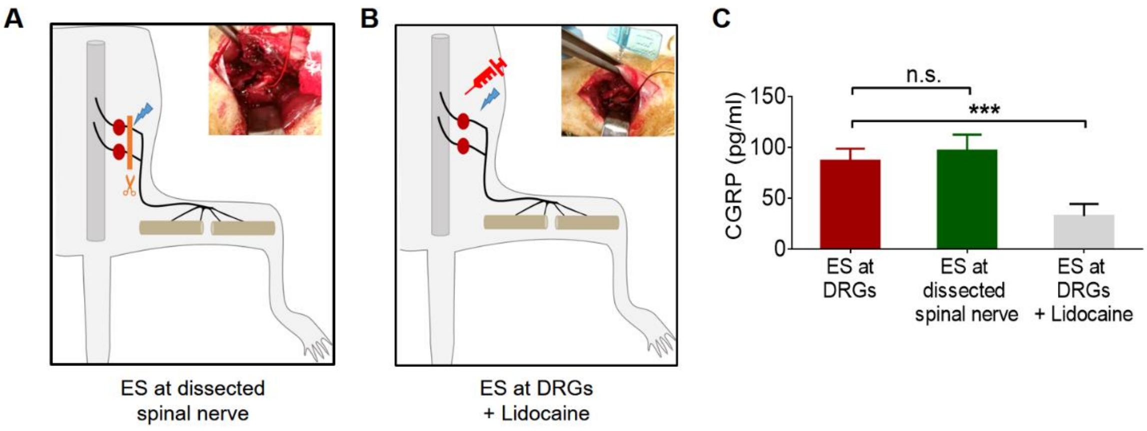

Figure S5. ES triggered the release of CGRP at femoral midshaft via action potential.

(**A**) Spinal nerve distally next to DRGs was dissected. (**B**) Lidocaine was continuously delivered around the stimulated DRGs via a syringe. (**C**) CGRP concentration in the femoral regions of ES at DRGs, ES at dissected spinal nerve, ES at DRGs + lidocaine group (mean ± SD, one-way ANOVA with *Tukey's* tests, ***$P < 0.001$, n.s. $P > 0.05$, $n = 4$ per group).

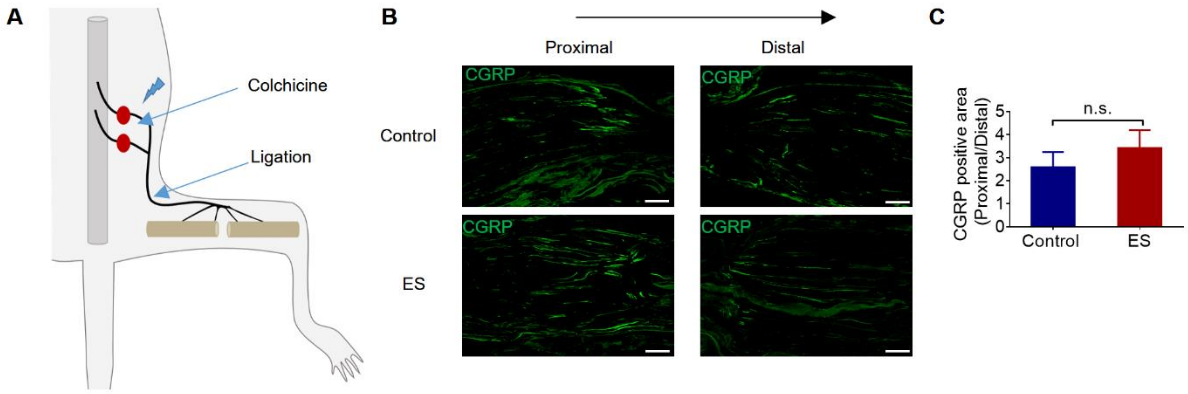

Figure S6. The axonal transportation of CGRP after ES.

(**A**) The sciatic nerve was ligated with suture. Colchicine was locally applied around DRGs during ES. Three hours after ES, sciatic nerve was collected for immunofluorescence analysis. (**B**) Representative and (**C**) quantification of CGRP positive area (proximal versus distal) of the ligated sciatic nerve of the control and ES group (mean ± SD, unpaired Student's *t* test, $n = 4$ per group, n.s. $P > 0.05$). Scar bar, 200 μm.

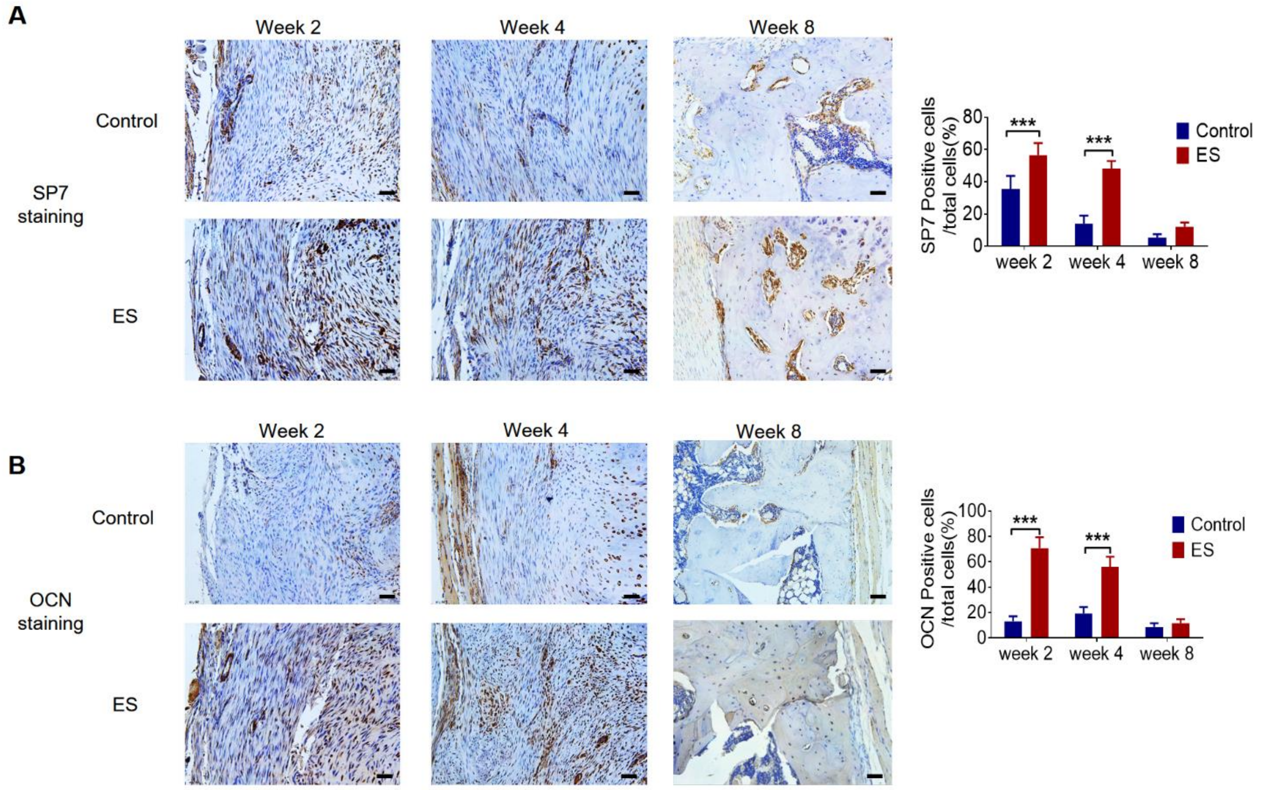

Figure S7. ES at DRGs increased SP7 and OCN expression at fracture callus.

(**A, B**) Immunohistochemical staining and quantification of SP7 and OCN positive cells at fracture callus in ES and the control group at week 2, 4 and 8 (mean ± SD, two-way ANOVA with *Bonferroni* tests, ***$P < 0.001$, $n = 5$ per group). Scale bar, 50 µm.

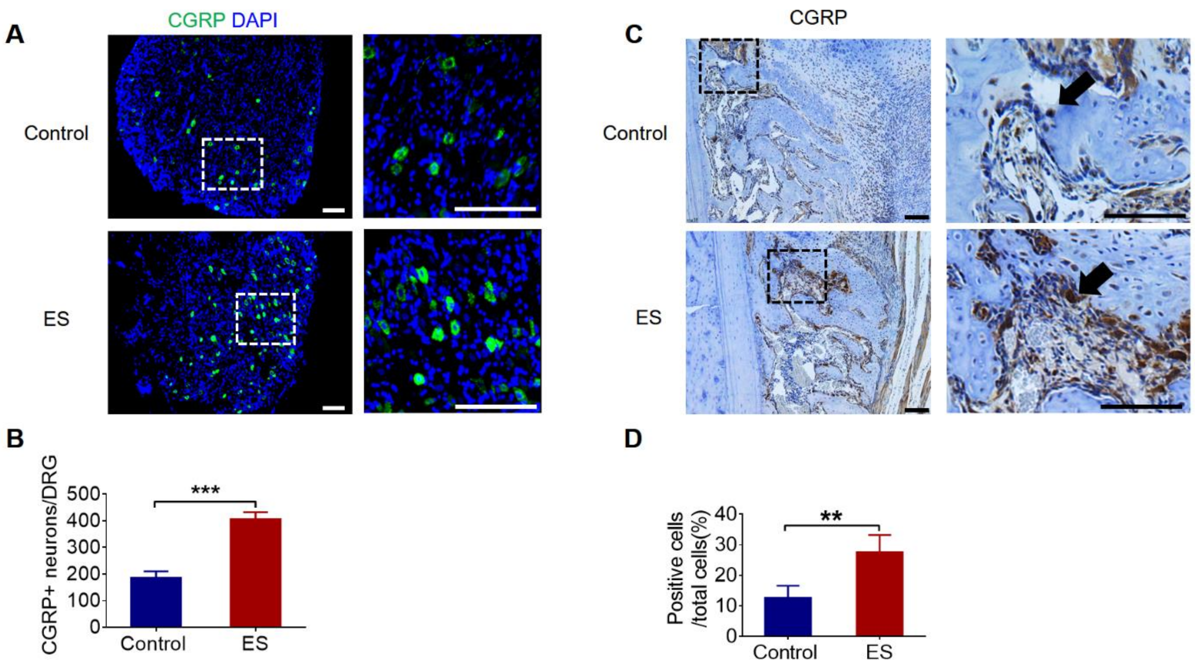

Figure S8. ES increased CGRP expression at DRGs and fracture callus in osteoporotic fracture.

(**A**) Representative fluorescence images and (**B**) quantification of CGRP positive neurons in the ipsilateral DRGs of both ES and the control group at week 2 (mean ± SD, unpaired Student's *t* test, ***$P < 0.001$, $n = 5$ per group). Scale bar, 100 µm. (**C**) Immunohistochemical staining and (**D**) quantification of CGRP positive cells (black arrows) at fracture callus in ES and the control group at week 2 (mean ± SD, unpaired Student's *t* test, **$P < 0.01$, $n = 5$ per group). Scale bar, 100 µm.

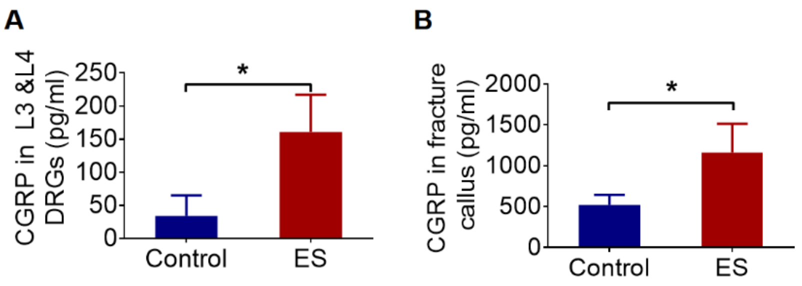

Figure S9. ES increased CGRP expression at DRGs and fracture callus in non-osteoporotic fracture.

(A) The CGRP concentration in L3 and L4 DRGs that were isolated and lysed with 500 μl RIPA at week 2. (B) The CGRP concentration in fracture callus that was collected and lysed with 30 μl mg^{-1} RIPA at week 2. Mean ± SD, unpaired Student's *t* test, *$P < 0.05$, $n = 3$ per group.

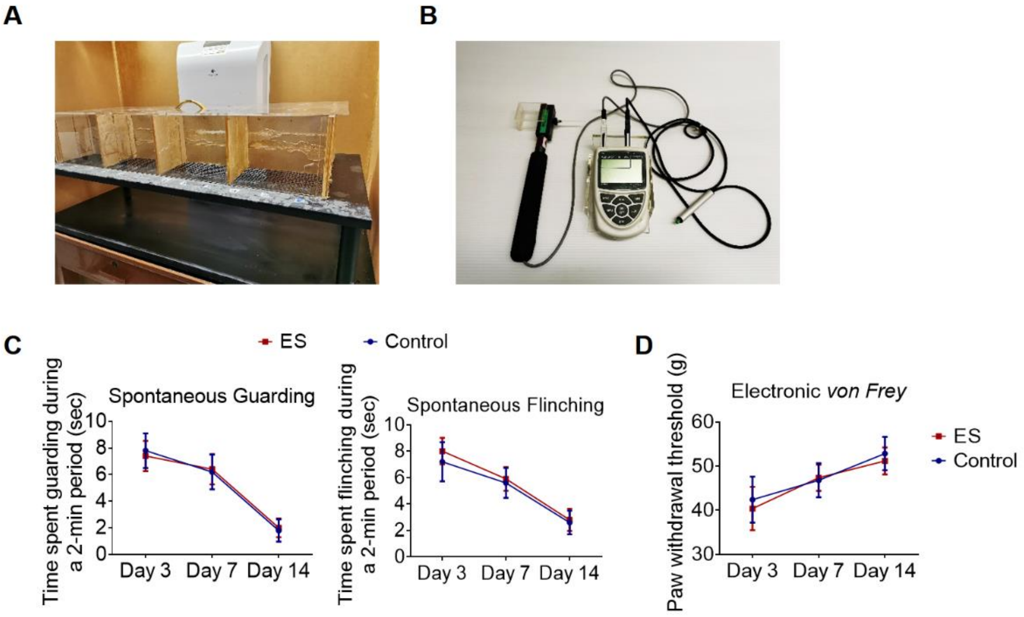

Figure S10. ES at DRGs did not elevate pain suffer.

(**A**) Separate cages with a perforated metal sheet and (**B**) electronic *von Frey* settings used for pain level assessments. (**C**) Spontaneously guarding and flinching from day 3 to day 14 after surgery (mean ± SD, repeated ANOVA with *Tukey's* test, *n* = 5 per group). (**D**) Paw withdrawal thresholds measured by electronic *von Frey* test from day 3 to day 14 after surgery (mean ± SD, repeated ANOVA with *Tukey's* test, *n* = 5 per group).

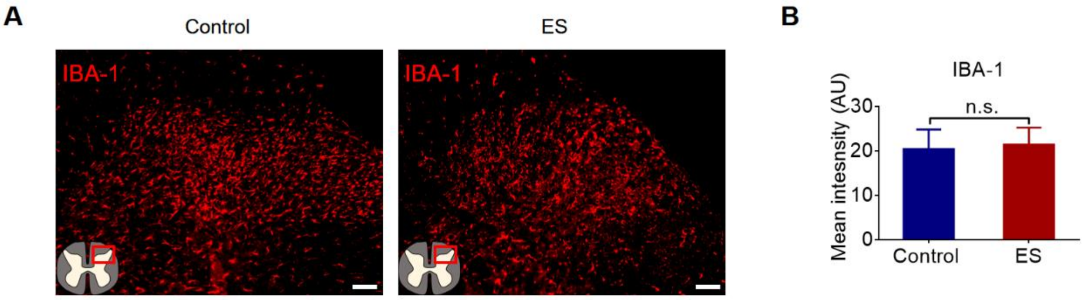

Figure S11. ES at DRGs did not evoke spinal microglial activation at week 2.

(**A**) Representative fluorescent images and (**B**) quantification of IBA-1 staining intensity in the ipsilateral dorsal horn segment (L3-L4) of ES and control group (mean ± SD, unpaired Student's *t* test, n.s. $P > 0.05$, $n = 5$ per group). Scale bar, 100 μm.

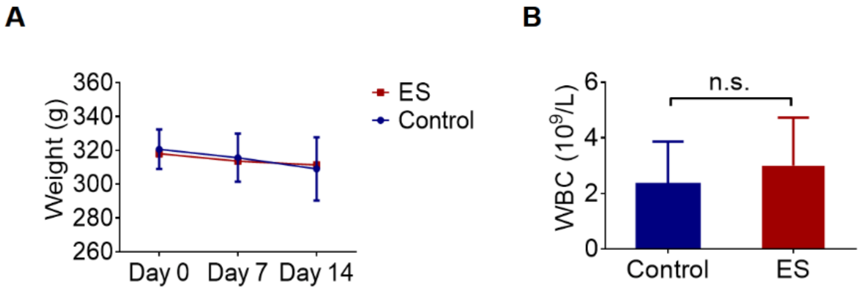

Figure S12. The body weight and white blood cell in peripheral blood cell after bioelectronic device implantation.

(A) Dynamic change of body weight (mean ± SD, two-way ANOVA with *Bonferroni* tests, $n = 3$ per group). (B) White blood cell (WBC, mean ± SD, unpaired Student's *t* test, $n = 3$ per group, n.s. $P > 0.05$).

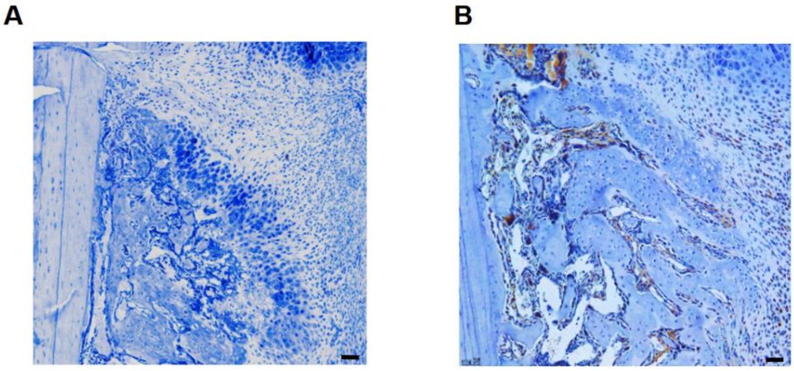

Figure S13. Representative isotype control and CGRP staining.

(A) Isotype control and (B) CGRP positive stained using IHC staining. Scale bar: 100 μm.

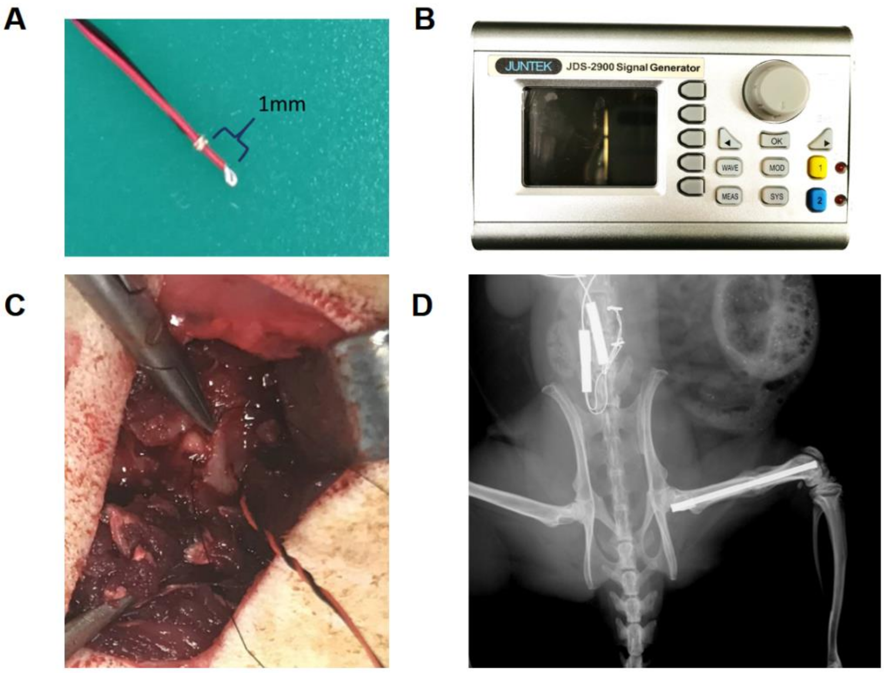

Figure S14. The bioelectrical system used in *in vivo* study.

(A) Electrodes and (**B**) stimulator used in current study. (**C**) A hole was drilled at transverse process for inserting an electrode at DRGs. (**D**) Electrodes remained their position after 2 weeks of daily stimulation.
